# Scoring Methods for Building Genotypic Scores: An Application to Didanosine Resistance in a Large Derivation Set

**DOI:** 10.1371/journal.pone.0059014

**Published:** 2013-03-21

**Authors:** Allal Houssaini, Lambert Assoumou, Veronica Miller, Vincent Calvez, Anne-Geneviève Marcelin, Philippe Flandre

**Affiliations:** 1 INSERM U943, Paris, France; 2 UMR-S 943, Université Pierre et Marie Curie (UPMC), Paris, France; 3 Forum for Collaborative Research, George Washington University, Washington, D.C., United States of America; 4 Virology Laboratory, Pitié-Salpêtrière Hospital, Paris, France; University of Pittsburgh, United States of America

## Abstract

**Background:**

Several attempts have been made to determine HIV-1 resistance from genotype resistance testing. We compare scoring methods for building weighted genotyping scores and commonly used systems to determine whether the virus of a HIV-infected patient is resistant.

**Methods and Principal Findings:**

Three statistical methods (linear discriminant analysis, support vector machine and logistic regression) are used to determine the weight of mutations involved in HIV resistance. We compared these weighted scores with known interpretation systems (ANRS, REGA and Stanford HIV-db) to classify patients as resistant or not. Our methodology is illustrated on the Forum for Collaborative HIV Research didanosine database (N = 1453). The database was divided into four samples according to the country of enrolment (France, USA/Canada, Italy and Spain/UK/Switzerland). The total sample and the four country-based samples allow external validation (one sample is used to estimate a score and the other samples are used to validate it). We used the observed precision to compare the performance of newly derived scores with other interpretation systems. Our results show that newly derived scores performed better than or similar to existing interpretation systems, even with external validation sets. No difference was found between the three methods investigated. Our analysis identified four new mutations associated with didanosine resistance: D123S, Q207K, H208Y and K223Q.

**Conclusions:**

We explored the potential of three statistical methods to construct weighted scores for didanosine resistance. Our proposed scores performed at least as well as already existing interpretation systems and previously unrecognized didanosine-resistance associated mutations were identified. This approach could be used for building scores of genotypic resistance to other antiretroviral drugs.

## Introduction

Antiretroviral therapy (ART) has significantly reduced the morbidity and mortality associated with human immunodeficiency virus (HIV) [1], [2]. Resistance to antiretroviral drugs is, however, a major factor limiting the effectiveness of ART [3], [4]. The drug resistance testing is an important monitoring tool and is recommended in clinical practice both when initiating therapy and to guide changes ART in patients with virologic failure [5], [6]. Interpreting the results of HIV-1 genotypic data is one of the most difficult task for clinicians caring for HIV-1 infected patients [7]. Three types of data form the basis of HIV-1 drug resistance knowledge: (1) correlations between viral genotype and ART of patients from whom sequenced HIV-1 isolates have been obtained (emergence of mutations under a specific-drug containing regimen); (2) correlations between genotype and phenotype (*in-vitro* drug-susceptibility test results); and (3) correlations between genotype, or phenotype, and virological response to a new treatment regimen.

Several interpretation systems (IS) have been developed to help choose appropriate ART in cases of resistance and three IS are currently used by clinicians: the French ANRS algorithm, the REGA algorithm and the Stanford HIV-db algorithm. ANRS and REGA algorithms combine virological data of types 1 and 2 and in many cases a genotypic score derived from a statistical analysis of resistance data (type 3). The HIV-db algorithm provides drug-specific estimates of viral susceptibility using a weighted scoring system for mutations thought to be associated with resistance. A more recent version of the HIV-db algorithm incorporates additional scores from combination rules, *i.e.*, presence of combinations of certain mutations. These systems are established by expert panels that review the current literature on drug resistance-associated mutations; their work involves correlating genotypic patterns with clinical data then translating the findings into interpretations rules [8]. These IS represent the sum of available knowledge and, are regularly updated, as necessary, for particular drugs. However, different IS give different results for some drugs [9]–[12].

The genotypic IS that are currently widely used may not perform satisfactorily for newly available data sets. The poor performance of scores and of existing IS emphasizes the need for improvements; new methods or larger data sets are required. It has also been shown that the variability observed in the different genotypic resistance scores was mainly due to the baseline patients characteristics than of the statistical methods used [13]. Cross-validation has been promisingly used as an attempt to overcome the problem of the lack of an external validation set [14]–[16] although external validation sets, from large cohorts, can also be used in some cases [17].

Many statistical methods, from simple linear models to more sophisticated approaches, have been proposed either to predict HIV-1 resistance from the genotype or phenotype [15], [18]–[22] or to identify resistance mutations associated with virological response [23]–[31]. For the latter, relevant resistance mutations are often grouped to give a genotypic score that can be included in existing IS (see above). Other works have investigated the performance of IS, or of newly derived scores, in the prediction of response to particular antiretroviral regimen [17], [32]–[34]. The question of whether a newly proposed methodology can improve predictions in the context of expert systems remains a subject of interest. Recently, various methods have been used to develop weighted genotypic scores based on regression models [17], [24]. This strategy involves using coefficients or coefficient transformation from different types of regressions as weighted scores for resistance.

The aim of this study was two-fold: i) to compare a small number of ‘scoring’ methods for building weighted genotypic scores, ii) to compare such scores with existing IS, in particular by using an external validation data set. These scores are used to classify patients as ‘resistant’ or ‘sensitive’ to the corresponding antiretroviral drug. We focus on scoring framework methodologies based on Linear Discriminant Analysis (LDA), Logistic Regression (LogReg), and Support Vector Machine (SVM). We will refer to strategies that involve classification by summing assigned weights to explanatory variables as ‘scoring strategies’. Such scoring strategies have not been extensively used for building algorithms for predicting HIV-1 resistance to antiretrovirals. The weights assigned are estimated through statistical methods in which the parameters and decision rules are directly available. It is well known that a score created from a particular data set will, with that data set, always outperform a score created using a different data set as there is always some degree of model overfitting. We therefore divided our data set into four country-based samples to investigate whether country-based scores provided better performance on external data sets than existing IS. As an illustrative example, we used the Forum for Collaborative HIV Research didanosine database [9]. Section 2 presents the data set used for this study, the Linear Discriminant Analysis, Logistic Regression and Support Vector Machine used in the scoring framework and the criteria for comparing the different systems. Results are presented in Section 3 and some elements of discussion are provided in Section 4.

## Materials and Methods

### Data set

We used the FORUM database for didanosine (ddI) based on 13 studies (clinical trials and clinic-based cohorts). Briefly, this database, which has been described elsewhere [9], [24], includes data for 1453 drug-experienced patients who had viral load >500 copies/mL and who underwent a genotypic resistance test when beginning a ddI-containing therapy. The median number of previously used antiretroviral drugs was four (range 1–12), including three NRTIs (0–6) and one protease inhibitor (0–4). Thirty-one percent (31%) of the patients had been pretreated with ddI before inclusion. The sample was split into four independent samples based on country of enrolment: France (n = 474), USA/Canada (219), Italy (440) and S/U/S (Spain/UK/Switzerland n = 320). The virological outcome was a change in HIV-1 RNA between baseline and week 8, and a response was defined as a HIV-1 RNA reduction of 0.6 log_10_ copies/ml or more. For building our scores we only investigated mutations detected in more than 1% of patients and providing a *p*-value <0.05 (Mann-Whitney test) for the association with the virological response. On the basis of these criteria 32 mutations were included in the study.

### Methods

A wide range of machine learning algorithms are currently available as off-the-shelf packages. We considered the following: Linear Discriminant Analysis (LDA), Support Vector Machine with linear separator (SVM), and Logistic Regression (LogReg). These methods provide: i) a set of coefficients associated with variables (*i.e*. mutations); and ii) a boundary decision. These can be used to classify patients into two groups (‘resistant’ or ‘sensitive’) based upon their individual scores: a score for a patient is the sum of the weights of the individual mutations present on virus genotype. The patient is then classified by comparing this score to the boundary decision. These methods use different mechanisms to search over the space of parameters: LDA estimates linear functions that maximize the distance between the categories, (*i.e.* an equation that has strong discriminatory power), and at the same time minimize the possibility of misclassifying cases into inappropriate groups or categories.In the basic setting, only one discriminant function is built (two groups *eg.* ‘resistant’ and ‘sensitive’). The boundary decision is based on the centroids of the groups. SVM methods find the best separation of input data, known as the optimal hyperplane which minimizes some criteria (see [35], [36] for more details). A linear separator is used in the case of two groups, the border is a linear function (known as a “Support Vector Regressor”). If there are two classes, the boundary decision is the position relative to a real number. For Logistic Regression, the set of parameters (β) is computed as usual and provide a linear predictor which is used to compute the probability of an ‘event’ in the model. Note that estimated coefficients associated with mutations reflect their importance for the resistant/sensitive classification. These approaches are complementary and can reveal different aspects of modeled scenarios. For a given sample, the three methods thus provide three independent scores leading to three different classifications.

Didanosine resistance IS were evaluated on the entire sequence for the RT region. The ANRS and REGA algorithm (version October 2011 and 8.0.2, respectively), HIV-db score system (version 29 May 2012) and a recent ddI algorithm developed with the same Forum data set [24] that we called LDVD (Large Derivation and Validation Data sets) were studied. ANRS, REGA and LDVD classify patients into three levels of inferred drug resistance: ‘sensitive’, ‘intermediate resistant’ and ‘resistant’. In order to compare these interpretations systems to our data-based scores, we investigates two aggregations: i) ‘intermediate’ and ‘resistant’ as ‘resistant’ (called in the following ANRS I, REGA I and LDVD I, respectively); ii) ‘sensitive’ and ‘intermediate’ as ‘sensitive’ (respectively ANRS II, REGA II and LDVD II, respectively). Adjustment of the cut-off score for the HIV-db algorithm is not as straightforward. This system provides five levels of inferred drug resistance (sensitive (S), potential low-level resistance (P), low-level resistance (L), intermediate resistance (I) and high-level resistance (R)). Thus, to compare this interpretation system, three different output normalization systems were used as follows: HIV-db I, S = ‘sensitive’ and P+L+I+R = ‘resistant’; HIV-db II, S+P = ‘sensitive’ and L+I+R = ‘resistant’; HIV-db III S+P+L = ‘sensitive’ and I+R = ‘resistant’.

The main difficulty in building a score from a data set is its validation. Such scores are generally highly data-dependent, and perform better than other scores or existing IS on the data set that was used to train it. In the absence of validation sets, cross-validation has been widely investigated as an approach for providing learners or models to establish robust rules for use in other populations of patients [14]–[16]. Although this procedure is valuable, the use of an external set, when possible, provides a better view of the performance of a score applied to independent populations. The data set used for this study is already subdivided into four subsets based on country of origin and therefore provides an opportunity to investigate external validation. The methods described above were applied to the four data sets providing such that four country-based scores were obtained for each statistical method. Each country-based score was applied to the data subset used to train it and then to the three other data subsets. We also compared performance of global scores and existing IS on the four country-based samples. Global scores for the whole data set are not independent of country-based data subsets but existing IS are also not independent of the studies included in the FORUM database. Data included in the FORUM database on didanosine were pooled from 13 trials or cohorts representing knowledge about didanosine resistance that was directly or indirectly used to build existing IS. For example, analysis of the Jaguar trial provided a genotypic score that was included in the ANRS algorithm [28].

Analyses aiming to improve the prediction of virological response, often use sensitivity, specificity (respectively defined as *true positive rate* and *1-false positive rate*) and the Receiver Operating Characteristic (ROC) curves. Different models can be compared by considering area under ROC curves with corresponding p-values [23], [33]. The models are designed to classify patients as being at low or high risk of response according to the set of variables included in the model. Here, we focus only on defining resistance classes based on genotype and no other variables are included in the three models used. If a patient is classified as ‘resistant’, it is predicted that he will have no virological response whatever the level of the other baseline variables (adherence, baseline viral load, etc.). If a patient is not defined as ‘resistant’, its virological response is strongly dependent of the values of other variables. We used the precision as the criterion for comparing the different methods; for this purpose, precision is defined as the number of patients classified as ‘resistant’ who indeed show no virological response (non-responder) as a proportion of the total number of patients classified as resistant.

## Results

**Figure 1:**
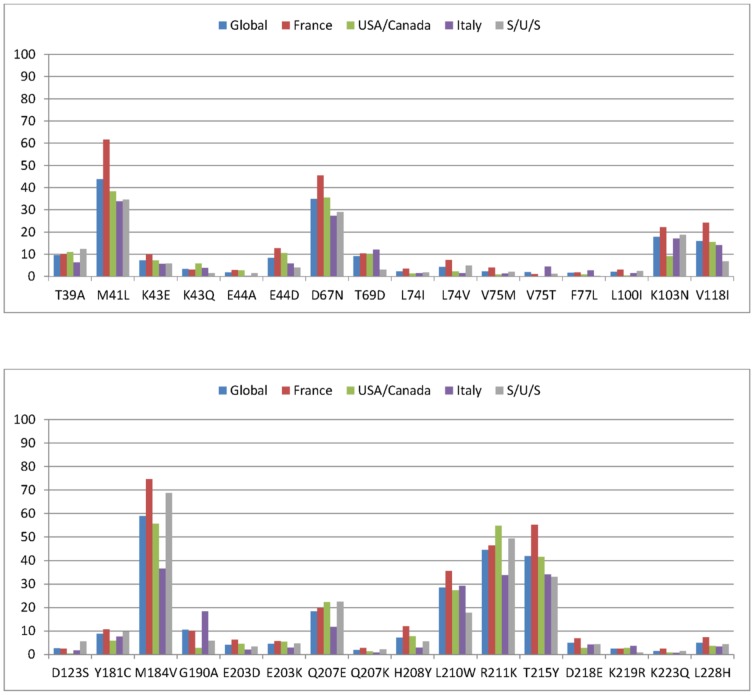
Frequency of the 32 mutations retained in the analysis on the entire data set and by country-based data sets.

The prevalence of each of the 32 mutations included in the analysis is shown in Figure 1. The most frequent mutations in the entire data set are: M184V (prevalence 58.9%), R211K (44.5%), M41L (43.7%), T215Y (41.9%), D67N (34.8%), L210W (28.5%), Q207E (18.4%), K103N (17.9%) and V118I (16.0%). Overall, the median number of mutations was 3 (interquartile range, 2–6) and 84 patients carried none of the 32 mutations included in the study. The French data set has the highest prevalence for seven of the nine mutations with prevalence higher than 15% (M41L, D67N, K103N, V118I, M184V, L210W and T215Y). Twenty-two of the 32 mutations were more prevalent in the French than in the other three data subsets Most of the patients were men (78%) and 995 (68.5%) patients were defined as ‘responders' (HIV-1 reduction greater than 0.6 log_10_ copies/mL), as previously reported [9]. Parameters and weights associated with the 32 mutations for global scores, based on the entire data set, are shown in Table 1. For LDA and SVM, sums of both intercept and weights are compared with cut-off values of 0 and 0.5, respectively, to classify patients as ‘resistant’ (>0 and >0.5, respectively). For the logistic regression, the linear predictor is determined by the parameters of the corresponding mutations and a patient is classified as ‘resistant’ if the corresponding probability is greater than 0.5. Rules for the SVM are simple a patient is classified as ‘resistant’ if the virus carries the L74I, F77L, or H208Y mutations. Of note, these three mutations are present in 2.3, 1.7 and 7.2% of patients in the global sample, respectively, and H208Y and L74I are more prevalent in France than in the three other samples. Classification for the two other methods is not so simple; nevertheless mutations F77L, Q207K and K223Q have the greatest weight in defining a patient as ‘resistant’. Discordance between SVM and both LogReg and LDA was higher than that between LogReg and LDA (Table 2). Overall, 103 patients were classified as ‘resistant’ by all three methods and 88 were classified as ‘resistant’ by LDA and LogReg but as ‘sensitive’ by SVM (Table 2).

**Table 1:** Estimated weights associated with the 32 mutations retained on the entire data set for the three statistical methods investigated (LDA: Linear Discriminant Analysis; LogReg: Logistic Regression, SVM: Support Vector Machine).

	*LDA*	*LogReg*	*SVM*
**Intercept**	−136	−1.25	−100
**Mutations**			
**T39A**	2	0.01	0
**M41L**	18	0.19	0
**K43E**	14	0.12	0
**K43Q**	−47	−0.42	0
**E44A**	−4	−0.06	0
**E44D**	−31	−0.30	0
**D67N**	27	0.26	0
**T69D**	31	0.26	0
**L74I**	85	0.77	167
**L74V**	44	0.40	0
**V75M**	−11	−0.09	0
**V75T**	72	0.62	33
**F77L**	121	1.08	167
**L100I**	41	0.38	0
**K103N**	14	0.13	0
**V118I**	30	0.27	0
**D123S**	99	0.87	0
**Y181C**	4	0.02	0
**M184V**	−47	−0.47	0
**G190A**	54	0.49	0
**E203D**	43	0.39	0
**E203K**	32	0.27	0
**Q207E**	35	0.34	0
**Q207K**	111	1.06	0
**H208Y**	66	0.57	200
**L210W**	67	0.61	0
**R211K**	0	0.00	0
**T215Y**	−15	−0.15	0
**D218E**	37	0.34	0
**K219R**	−29	−0.25	0
**K223Q**	102	0.94	0
**L228H**	27	0.24	0

**Table 2:** Classification as ‘resistant’ or ‘sensitive’ according to the three statistical methods investigated on the entire data set (LDA: Linear Discriminant Analysis; LogReg: Logistic Regression, SVM: Support Vector Machine).

Method			
LDA	LogReg	SVM	N
Resistant	Resistant	Resistant	103
Resistant	Resistant	Sensitive	88
Resistant	Sensitive	Resistant	6
Resistant	Sensitive	Sensitive	4
Sensitive	Sensitive	Sensitive	1207
Sensitive	Sensitive	Resistant	43
Sensitive	Resistant	Sensitive	2
Sensitive	Resistant	Resistant	0

The precision of each of the three methods and existing IS applied to the global dataset are reported in Table 3. Overall 201, 193 and 152 patients were classified as ‘resistant’ with the LDA, LogReg and SVM methods, respectively. As expected, the scoring methods performed better than already existing IS because the global data set was used both for training and validating these scores. Scoring methods classified approximately 60% of the patients without virological response at week 8 as ‘resistant’ with no significant difference between the three methods. The precision of existing IS was, on average, of 40% (range 37% for HIV-db I to 58% for LDVD II). LDVD II, however, classified only 67 patients as ‘resistant’ such that the confidence in this the precision value was poor (58.2%, 95% confidence interval 45.5 to 70.2%). It appears that there is a correlation between the precision and the number of patients classified as ‘resistant’ since the ANRS I and II provided a good performance (49%) with approximately 370 patients classified as resistant while HIV-db I provided the poorest performance (37%) with more than 1000 patients defined as ‘resistant’

**Table 3:** Number of patient with a virological response or not among patients classified as ‘resistant’ and precision for the three statistical methods and existing IS on the entire data set (LDA: Linear Discriminant Analysis; LogReg: Logistic Regression, SVM: Support Vector Machine).

	Patients classified as ‘resistant’			
	Responder (n)	Non Responder (n)	Total (n)	Precision (%)
Method				
*LDA*	80	121	201	60.2
*LogReg*	75	118	193	61.1
*SVM*	61	91	152	59.9
Existing IS				
ANRS I	195	184	379	48.5
ANRS II	184	174	358	48.6
LDVD I	355	274	629	43.6
LDVD II	28	39	67	58.2
REGA I	560	337	897	37.6
REGA II	216	153	369	41.5
HIV-db I	650	376	1026	36.6
HIV-db II	590	356	946	37.6
HIV-db III	498	313	811	38.6

Global scores and existing IS were also applied to each country-based data set (Table 4). Precision was generally much better for scoring methods than for existing IS. Weighted means of the precision were around 60% for scoring methods and were between 37 and 49% for existing IS (except for LDVD II but, again, with a large 95% confidence interval as explained above). The French data set provided the best performance, and this was a consequence of the high prevalence of most of the mutations contributing to global scores and used by existing IS. For the three other subsamples, the precisions were better than or similar to those for any existing IS. The SVM method performed better than the two other methods for the Italian subsample, but the inverse was observed for the S/U/S subsample. There was no significant difference between the three statistical methods for French and USA/Canadian data subsets. The poorest performance (between 13 to 39%) with existing IS was for the S/U/S data subset.

**Table 4:** Precision for global scores, obtained from the three statistical methods, and existing IS for each country-based data sets (S/U/S: Spain/UK/Switzerland; LDA: Linear Discriminant Analysis; LogReg: Logistic Regression, SVM: Support Vector Machine).

	Precision (%)				
	France	USA/Canada	Italy	S/U/S	Weighted mean
Method					
LDA	75.8	45.8	44.1	55.6	60.2
LogReg	78.2	45.8	42.9	57.7	61.1
SVM	74.4	42.9	53.3	34.8	59.9
Existing IS					
ANRS I	62.5	42.9	40.3	33.9	48.5
ANRS II	62.0	44.7	36.7	38.5	48.6
LDVD I	56.3	41.4	42.0	17.5	43.6
LDVD II	84.6	66.7	38.5	16.7	58.2
REGA I	47.8	35.5	38.5	15.2	37.6
REGA II	58.7	34.0	37.9	13.0	41.5
HIV-db I	45.6	34.1	39.2	16.0	36.6
HIV-db II	46.7	36.1	39.2	16.9	37.6
HIV-db III	49.8	38.2	37.2	15.6	38.6

The three methods were applied to the four country-based data subsets providing four country-based scores for each method. Intercepts and weights specific to each country-based subsample are shown in Table S1. Scores were applied to all country-based data subsets: one subset was used to build the score which was then applied to the three other subsets (Table 5). Considering four blocks representing the four data sets, the set used for both training and validating the score is displayed in the first row of each block. For each method, the precision obtained from country-based data subsets was better than that with the global data set (the weighted mean of precision computed from the four country-based data sets is higher than the precision obtained from the global data set). For instance, the global precision for LDA was 60.2% (Table 3) whereas the weighted mean obtained from the four country-based data subsets was 68.8% (weighted mean based on 72.9, 73.5, 57.4 and 68.3%, Table 5).

**Table 5:** Precisions of country-based scores for each country-based data sets including the sample that served for training (S/U/S: Spain/UK/Switzerland; LDA: Linear Discriminant Analysis; LogReg: Logistic Regression, SVM: Support Vector Machine).

		Precision		
		LDA	LogReg	SVM
		**Validation set: France**		
**Training**	**France**	72,9	72,0	72,0
	**USA/Canada**	53,1	48,8	53,5
	**Italy**	68,4	66,7	66,7
	**S/U/S**	63,2	64,9	60,4
		**Validation set: USA/Canada**		
**Training**	**USA/Canada**	73,5	72.7	79,2
	**France**	45,5	45,7	42,3
	**Italy**	36,7	30,8	31,6
	**S/U/S**	28,6	25,0	25,0
		**Validation set: Italy**		
**Training**	**Italy**	57,4	39,6	63,6
	**France**	45,9	45,8	45,0
	**USA/Canada**	44,0	38,7	38,7
	**S/U/S**	44,4	45,7	45,2
		**Validation set: S/U/S**		
**Training**	**S/U/S**	68,3	77,8	66,7
	**France**	31,7	30,9	31,1
	**USA/Canada**	28,6	28,5	25,0
	**Italy**	29,4	28,1	33,3
	**Weighted Mean**	68,8	69,7	70,8

External validation provided the lowest values of precision. For example, the country-based score trained with the Italian subsample and applied to the USA/Canada subsample led to precisions of 36.7, 30.8 and 31.6 for LDA, LogReg and SVM, respectively, whereas when the USA/Canada data set was used for both training and validating the values were 73.5, 72.7 and 72.0, respectively (Table 5). The difference in precision, however, was much larger when the validating set was USA/Canada and S/U/S than when it was France and Italy. Nevertheless, the performances of country-based scores applied to an external sample were better than or similar to those of existing IS. For example, the performance of country-based scores using the S/U/S sample as the validating set was between 25 and 33.3% (Table 5) and for existing IS it was between 13 and 38.5% (Table 4).

## Discussion

The choice of treatment for HIV-infected patients with virological failure is of major importance in their management. Expert-based IS are valuable tools for choosing the appropriate combination therapy for such patients [7]. However, different IS give different results for certain drugs leading to potential confusion among clinical practitioners [9], [11], [12]. The process in these IS by which weights are associated with mutations has not been clearly defined. The aim of this analysis was to use a large data set to assess three methods for providing weighted scores that can be used to define resistance class from genotype information. The total sample was divided into four country-based subsamples to allow external validation of global scores and country-based scores. Resistance to didanosine was used to illustrate the methodology which should be applied for more recent antiretroviral drugs.

Using the global data set for training and validating, all three scoring methods performed better than currently exiting IS. This was expected, because scores generated on a particular sample perform better than any other rules on that sample. We did not find any difference in precision between the three methods, although the SVM method gave a classification that was not the same as those given by the LDA and LogReg methods. Global weights indicated that the SVM classification was based on only three mutations and suggest that LDA and LogReg are more appropriate. With SVM, weights obtained from country-based data sets, however, provided a different finding because many mutations had a non-zero weights, except in the Italian subsample (Table S1). The reasons why many weight estimates were zero in the global and Italian data sets is unclear and needs further investigation. Logistic regression is a simple method, available in many statistical software packages, and has been successfully used in the context of models of resistance to antiretrovirals [32], [33].

For the entire sample, ANRS, LDVD and REGA II provided better precision than HIV-db, contradicting previous results for didanosine resistance based on the FORUM database [9]. However, our criterion was very different to those used previously in predictive models: we focused on the precision measures as the ability to predict resistance from the genotype and then to predict virological failure among patients classified as resistant. It appears that there is a negative correlation between the number of patients classified resistant and precision. The HIV-db IS gives the largest number of patients classified as ‘resistant’ because it considers mutations at 21 positions to assess didanosine resistance, the ARNS and REGA systems include only 9 and 11 positions, respectively.

Scores defined from the entire sample and applied to country-based data subsets performed better than already existing IS. As each country-based sample is involved in building the global scores, the global scores could be considered not to be independent of the country-based data subsets. This is also true, however, for existing IS. Indeed, the FORUM database on didanosine contains some of the data used to build these IS. For example, the didanosine score defined in the Jaguar study has been included in the ANRS IS since 2007. The studies included in the FORUM database represent a part of the knowledge about didanosine resistance that was used to define some of the rules in existing IS. We provide a comparison of different rules applied to four distinct samples in Table 4. The poor performance of the S/U/S subset is explained because data from three different countries are included in this subset. The best performance was with the French subset because this data set has the highest prevalence of 22 mutations and high prevalence of the major mutations (L74I, F77L, D123S, Q207K, H208Y, L210W and K223Q) involved in the three scoring systems. It has been shown that the France data set includes the largest number of patients previously exposed to didanosine and the highest median number of previous antiretroviral drugs [13].

Country-based scores applied to country-based samples used to build them showed greater precision than global scores applied to the global data set. This is because country-based subsamples provided more specific scores due to a smaller variability between patients than in the global data set. Conversely, scores defined on one country-based data set showed lower precision when applied to another data set, as observed previously [9], [17]. This limitation is inherent to scores defined on one particular population and applied to completely independent samples. There were substantial differences in the prevalence of the various mutations between the four country-based data sets (Figure 1). These epidemiological differences are probably consequences of differences in treatment histories between populations in different countries [13] although there may be other causes [17]. The precision was up to 45% lower when the USA/Canada and S/U/S data sets were used for validation. The smallest loss was when the score was trained on the Italian data subset and applied to the French data subset; we have no clear explanation for this. Despite difference in weights, especially between SVM and the two others methods, there was no significant difference in precision between the three statistical methods investigated.

Overall, seven mutations (L74I, F77L, D123S, Q207K, H208Y, L210W and K223Q) have a major impact on the resistance by at least one of the three scoring methods used. The L74I, and to a lesser extent the L210W and F77L mutations, are used by one or more existing IS to predict resistance. These IS do not use the four other mutations to predict didanosine resistance. The prevalence of these mutations is low, and this may explain why they were not found to be associated with didanosine resistance in previous studies based on small sample size. In the Stanford HIV Drug Resistance Database (http://hivdb.stanford.edu/cgi-bin/MutPrevBySubtypeRx.cgi), the percentages of viruses with the D123S, Q207K, H208Y and K223Q mutations in antiretroviral-experienced patients infected with subtype B are 0.8, 1.1, 3.6 and 0.7%, respectively. The D123S and Q207K mutations were already identified as being associated with didanosine resistance in the LDVD algorithm developed on the FORUM database [24]. Some mutations (insertion at codons 66, 67, 68, 69, 70 or 71, Q151M) used by existing IS as strong predictors of resistance to didanosine were not retained in our analysis because their prevalence is lower than 1%, and consequently they have no impact on resistance in our analysis. This emphasizes an apparent discordance between weights obtained from methods investigated here and weights in existing IS. Such a difficulty has been sometimes circumvented in including well known resistance mutations in a score even if there were not present in the sample [24], [30].

Few mutations had positive impact on virological response in our analysis and had negative impact in existing IS. For example, T215Y is considered to be a resistance mutation in the three IS investigated, but is scored by the LDA and LogReg analyses as having a positive effect. This discordance is explained by interactions with other mutations: the effect of each mutation on resistance is adjusted for the 31 other mutations in the model. For example, applying a logistic regression model after removing the seven mutations described above leads to the T215Y mutation being scored as having a negative effect (β = 0.04). This emphasizes both the complex pattern of mutations and the possibly different effects of each mutation in the presence or absence of other mutations, especially when the prevalence of some mutations is low. Resistance studies using ultrasensitive assays, including deep sequencing, will provide further insights into effects of this type. Discordant findings for the M184I/V mutation, even between existing IS, have been reported previously and discussed [28].

One limitation of this work is that the scoring methods investigated use a binary outcome. Moving from a continuous outcome, the HIV-1 RNA reduction, to a binary outcome, response versus no response, generally leads to a loss of information. The threshold of 0.6 log_10_ copies/mL used to define a virological response was chosen due to the moderate potency of didanosine. Sensitivity analyses, however, using 0.5 and 0.7 log_10_ copies/mL provided similar results (data not shown). Another limitation is the absence or low prevalence of certain mutations involved in resistance to NRTI-class. Some relevant mutations and insertions were absent from our database even though it is large. One consequence of the way that statistical models assess weights for resistance mutations adjusted for the presence of other mutations is that different weights are likely to be found in the presence of other mutations. This problem is inherent to the use of statistical methods. The good performance of global scores, however, with the four country-based data sets demonstrates the robustness of these methods. External validation of country-based scores confirm this and showed better performance than existing IS applied on the country-based samples. In conclusion, we explored a small number of scoring methods used to build weighted genotyping scores and to classify patients as resistant or not. The three methods performed similarly, and as well as or betterthan existing IS. Four mutations not previously shown to be associated with didanosine resistance were identified in our scores as having an impact. The prevalence of the four mutations in our large data set was low. In this study, we used didanosine as an illustrative example; these scoring methods should be applied to resistance to other antiretroviral, particularly those that have been introduced recently, to assess their utility.

## Supporting Information

Table S1 Estimated weights associated with the 32 mutations for country-based scores obtained from the three statistical methods investigated (LDA: Linear Discriminant Analysis; LogReg: Logistic Regression, SVM: Support Vector Machine).
(DOCX)
