# Supplementary material for: Scoring Methods for Building Genotypic Scores: An Application to Didanosine Resistance in a Large Derivation Set
Source: PLoS One. 2013 Mar 21;8(3):e59014. doi: 10.1371/journal.pone.0059014 (PMC3605419; doi:10.1371/journal.pone.0059014)
Supplement: Table S1 — Estimated weights associated with the 32 mutations for country-based scores obtained from the three statistical methods investigated (LDA: Linear Discriminant Analysis; LogReg: Logistic Regression, SVM: Support Vector Machine). (DOCX) [file pone.0059014.s001.docx]

| **Data set** | **France** | | | **USA/Canada** | | | **Italy** | | | **S/U/S** | | |
| --- | --- | --- | --- | --- | --- | --- | --- | --- | --- | --- | --- | --- |
|  | *LDA* | *LogReg* | *SVM* | *LDA* | *LogReg* | *SVM* | *LDA* | *LogReg* | *SVM* | LDA | LogReg | *SVM* |
| Intercept | -175 | -1,58 | -126 | -108 | -0,91 | -100 | -109 | -1,03 | -100 | -226 | -1,59 | -104 |
| T39A | 35 | 0,28 | 29 | 102 | 1,01 | 33 | 20 | 0,17 | 0 | -66 | -0,53 | -39 |
| M41L | 3 | 0,05 | 7 | 75 | 0,78 | 47 | -33 | -0,34 | 0 | 57 | 0,68 | 8 |
| K43E | -2 | -0,06 | -23 | -123 | -1,27 | -30 | 98 | 0,96 | 100 | 14 | 0,23 | 0 |
| K43Q | 15 | 0,11 | 35 | -27 | -0,42 | 13 | -95 | -0,92 | 0 | -202 | -15,55 | -85 |
| E44A | -56 | -0,47 | -14 | 95 | 0,47 | 51 | 30 | 11,46 | 0 | 130 | 1,28 | 8 |
| E44D | -87 | -0,9 | -43 | 93 | 0,65 | 80 | -15 | -0,12 | 0 | -24 | -0,49 | 8 |
| D67N | 43 | 0,4 | 7 | 69 | 0,75 | 43 | 6 | 0,04 | 0 | -1 | -0,21 | 0 |
| T69D | 40 | 0,35 | 53 | -26 | -0,26 | -30 | 11 | 0,11 | 0 | -117 | -1,06 | -13 |
| L74I | 147 | 1,55 | 126 | -280 | -17,63 | -77 | 29 | 0,31 | 0 | 338 | 2,5 | 84 |
| L74V | 10 | 0,1 | 7 | -169 | -1,66 | -81 | -58 | -0,57 | 0 | 214 | 1,59 | 52 |
| V75M | 40 | 0,35 | 37 | -288 | -19,19 | -100 | 14 | 0,17 | 0 | 8 | 0,72 | -59 |
| V75T | 149 | 1,63 | 100 | 0 | 0 | 0 | 67 | 0,62 | 0 | -99 | -1,12 | 14 |
| F77L | -7 | 0,16 | 0 | -1 | -15,53 | -30 | 125 | 1,1 | 200 | 849 | 19,25 | 100 |
| L100I | 116 | 1,02 | 100 | -375 | -17,8 | -10 | 15 | 0,16 | 0 | 210 | 1,19 | 30 |
| K103N | 34 | 0,33 | 31 | 17 | -0,08 | 4 | -48 | -0,52 | 0 | 28 | 0,24 | 4 |
| V118I | 14 | 0,16 | 22 | 2 | 0,08 | -16 | 36 | 0,37 | 0 | -27 | -0,26 | 0 |
| D123S | 132 | 1,19 | 109 | 512 | 19,05 | 100 | 111 | 0,96 | 100 | 59 | 0,57 | 4 |
| Y181C | -27 | -0,24 | -19 | -41 | -0,13 | -13 | -11 | -0,09 | 0 | 167 | 1,1 | 44 |
| M184V | -24 | -0,26 | -12 | -157 | -1,55 | -76 | 2 | 0,03 | 0 | -84 | -0,86 | -4 |
| G190A | 143 | 1,38 | 140 | -19 | -0,51 | -37 | 33 | 0,32 | 0 | 4 | 0,04 | -9 |
| E203D | 3 | 0,05 | 0 | 269 | 3,01 | 90 | 34 | 0,34 | 0 | 128 | 1,21 | 8 |
| E203K | 77 | 0,81 | 51 | 25 | 0,58 | -13 | 70 | 0,75 | 0 | 125 | 1,21 | 4 |
| Q207E | 57 | 0,55 | 31 | 8 | 0,16 | 13 | 38 | 0,39 | 0 | 48 | 0,47 | 5 |
| Q207K | 75 | 0,87 | 38 | -92 | -15,7 | 0 | 44 | 0,43 | 0 | 405 | 2,59 | 100 |
| H208Y | 97 | 1,05 | 137 | -43 | -0,78 | -17 | -18 | -0,27 | 0 | -110 | -2,34 | 9 |
| L210W | 58 | 0,53 | 30 | 139 | 1,58 | 50 | 83 | 0,77 | 0 | 23 | 0,39 | 8 |
| R211K | 74 | 0,68 | 31 | -55 | -0,56 | -13 | 4 | 0,03 | 0 | -57 | -0,6 | -1 |
| T215Y | 5 | 0,01 | 0 | -43 | -0,72 | -21 | 4 | 0,07 | 0 | -63 | -0,95 | -21 |
| D218E | 16 | 0,08 | 21 | -14 | 0,01 | 0 | -26 | -0,24 | 0 | 130 | 1,06 | 16 |
| K219R | 34 | 0,36 | 13 | 95 | 0,55 | 77 | -81 | -0,9 | 0 | 345 | 3,67 | 100 |
| K223Q | 90 | 1,23 | 64 | -43 | -0,53 | 0 | 212 | 2,23 | 100 | 148 | 1,54 | 41 |
| L228H | 54 | 0,45 | 49 | -22 | 0,23 | 13 | -141 | -1,63 | -100 | 187 | 1,25 | 53 |

Table S1: Estimated weights associated with the 32 mutations for country-based scores obtained from the three statistical methods investigated (LDA: Linear Discriminant Analysis; LogReg: Logistic Regression, SVM: Support Vector Machine).
